# Analysis of the distribution of assimilation products and the characteristics of transcriptomes in rice by submergence during the ripening stage

**DOI:** 10.1186/s12864-018-5320-7

**Published:** 2019-01-08

**Authors:** Hyeon Seok Lee, Woon Ha Hwang, Jae Hyeok Jeong, Seung Hyeon Ahn, Jeong Seon Baek, Han Yong Jeong, Hong Kyu Park, Bon Il Ku, Jong Tak Yun, Geon Hwi Lee, Kyung Jin Choi

**Affiliations:** Crop Production & Physiology Division, National Institute of Crop Science, Rural Development Administartion, Wanju, 55365 South Korea

**Keywords:** Submergence, Rice, RNA-sequencing, Physiology, Ripening stage

## Abstract

**Background:**

Research on the submergence stress of rice has concentrated on the quiescence strategy to survive in long-term flooding conditions based on Submergence-1A (SUB1A). In the case of the ripening period, it is important that submergence stress can affect the quality as well as the survival of rice. Therefore, it is essential to understand the changes in the distribution of assimilation products in grain and ripening characteristics in submergence stress conditions. However, such studies have been insufficient at the physiological and molecular biological levels.

**Results:**

We confirmed that the distribution rate of assimilation products in grain was decreased by submergence treatment. These results were caused by an increase in the distribution rate of assimilation products to the stem according to escape strategy. To understand this phenomenon at the molecular level, we analyzed the relative expression levels of genes related to sucrose metabolism, and found that the sucrose phosphate synthase gene (OsSPS), which induces the accumulation of sucrose in tissues, was decreased in the seeds and leaves, but not in the stems. Furthermore, the sucrose transporter gene (OsSUT) related to sucrose transport decreased in the seeds and leaves, but increased in stems. We also analyzed the biological metabolic processes related to starch and sucrose synthesis, carbon fixation, and glycolysis using the KEGG mapper with selected differentially expressed genes (DEGs) in seeds, stems, and leaves caused by submergence treatment. We found that the expression of genes for each step related to starch and D-glucose synthesis was down-regulated in the seeds and leaves but up-regulated in the stem.

**Conclusion:**

The results of this study provide basic data for the development of varieties and corresponding technologies adapted to submergence conditions, through understanding the action network of the elements that change in the submergence condition, as well as information regarding useful DEGs.

**Electronic supplementary material:**

The online version of this article (10.1186/s12864-018-5320-7) contains supplementary material, which is available to authorized users.

## Background

Rice is one of the most important crops in the world and it is crucial for the food security of many Asian countries [1]. Recently, an increase in the occurrence of abnormal weather phenomena and progressive global warming caused by the accumulation of greenhouse gases have resulted in damage to the growth and development of rice plants, which threatens food security [2]. Among the abnormal weather phenomena, the occurrence of heavy rainfall, exceeding the predicted maximum rainfall based on past weather data, is expected to increase the occurrence of submergence of agricultural land and seriously reduce the production of rice [3].

It is known that photosynthesis and respiratory metabolism are inhibited when the plant is submerged because gas diffusion is reduced by more than 10,000-fold relative to that which occurs in the atmosphere, and light reaching the submerged leaves is attenuated by water [4, 5]. When rice is completely submerged to the ends of its leaves and the supply of oxygen from the atmosphere has stopped, the pyruvic acid produced by glycolysis is not used as a substrate for normal aerobic respiration through the tricarboxylic acid (TCA) cycle, but rather as an anaerobic respiration substrate, such as alcohol fermentation, in which pyruvic acid is converted to ethanol by pyruvate decarboxylase and alcohol dehydrogenase [6]. In the case of anaerobic respiration, one molecule of glucose produces only approximately 2 ATPs compared with approximately 38 ATPs produced by aerobic respiration, resulting in an energy shortage because the respiratory substrate is quickly consumed [7, 8]. If the anaerobic condition continues, the consumption of carbohydrates, such as monosaccharides and polysaccharides as the primary respiratory substrate is greatly increased, and protein is decomposed as a secondary respiratory substrate, such that it is greatly damaged or destroyed [9, 10]. In addition, submergence can cause the degradation of chlorophyll, decrease of Rubisco activity, and damage to the photosynthetic apparatus, resulting in a large reduction in photosynthesis [11, 12]. During the ripening period, such excessive consumption of the respiratory substrate and decrease in production and supply of assimilation products, may reduce the ripening rate, head rice ratio, and grain weight, which may result in decreased yield and quality.

Studies on submergence of rice have been conducted on a variety of topics, such as the quiescent strategy by the SUB1 gene, avoidance strategy by the SNORKEL 1 and 2 genes [8, 13], anaerobic respiration mechanism, hormone response [14], and remobilization of stored carbohydrates [15, 16]. Primarily studies on the SUB1 gene, which regulates the submergence resistance characteristics and was first found in the submergence-resistant FR13A variety, have been performed [17–19]. Recently, to develop a new breeding program, efforts have been made to discover another quantitative trait locus (QTL) and various genes that could produce new effects in combination with the submergence resistance characteristics of the SUB1 gene [20]. Some intermediate tolerant varieties, such as IR64, carry the allelic variant SUB1A-2 and its derived tolerance near the isogenic line IR64-Sub1, which carries the SUB1A-1 allele, were used to compare the expression of nearly 2500 rice TF genes [21]. Additionally, paclobutrazol, which could significantly enhance rice seedling survival in submergence conditions by retaining a higher level of chlorophyll content and alcohol dehydrogenase activity, and decelerating the consumption of non-structure carbohydrate, was treated with rice. In total, 3936 differentially expressed genes(DEGs), which could enhance the submergence tolerance, were identified by transcriptomic analysis [22]. RNA-seq analysis was conducted in order to identify transcriptome characteristics for the difference in early growth between submergence tolerant and sensitive varieties of rice during the germination stage in submergence conditions. Candidate DEGs, such as OsTPP7, HXK7, and PGM, which could affect early growth under submergence conditions were selected [23]. Like these studies, transcriptome analysis studies related to submergence stress have been conducted recently. However, there is insufficient research at the physiological and molecular levels on this subject during the ripening stage.

In this study, we analyzed the effects of submergence treatment on the distribution characteristics of assimilation products during the ripening stage, and selected the useful genes that exhibited a difference in expression in the seed, stem, or leaf organs. In addition, basic information that could be used to develop material and DNA markers for breeding adapted varieties with submergence resistance characteristics by precisely identifying the genetic factors that characterize the necessary traits during the ripening stage.

## Results

### Distribution of assimilation products caused by submergence treatment during the ripening period

Nonstructural carbohydrates (NSCs), such as starch and soluble sugar, accumulated in the stem and leaf sheath before the heading date, and these NSCs accounted for approximately 30% of the energy source during ripening after heading [24, 25]. In particular, when photosynthetic efficiency is decreased under environmental stress conditions, such as drought and salt stress, the use of the stored NSCs before heading would be an even more important factor in ripening [26]. In addition, the supply of oxygen to the plant was not smooth under the submergence condition, such that anaerobic respiration occurred, which caused energy production efficiency to drop sharply and eventually resulted in excessive consumption of respiration substrates (carbohydrates) [27, 28].

**Fig. 1:**
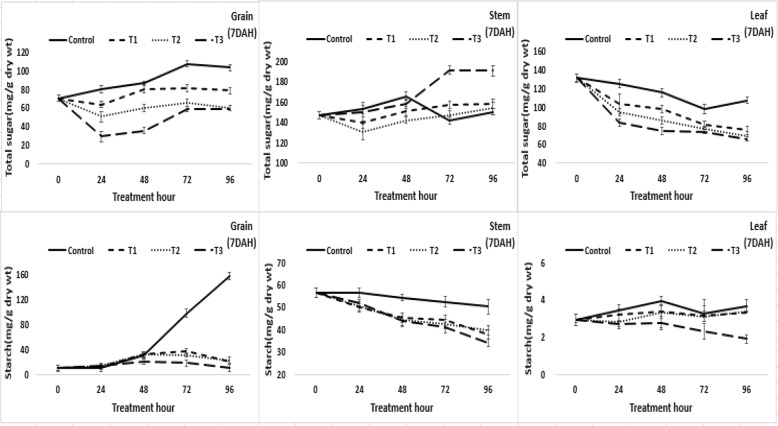
Changes in the soluble carbohydrate content. The changes in soluble carbohydrate content(total sugar and starch content) in grain, stems, and leaves affected by the flooding treatment hour at 7 days after heading(DAH). The treatment was carried out for up to 96 h and the samples were used in five replicates. T1: overheading and clear water flooding condition. T2: flag leaf exposure and muddy water flooding condition. T3: overheading and muddy water flooding condition

The changes in soluble carbohydrate content in grain, stems, and leaves affected by the flooding treatment at 7 days after heading are shown in Fig. 1. First, in grain, although starch synthesis started at approximately 7 days after heading and rapidly increased at approximately 8 days after heading in the control, starch synthesis was inhibited in all submergence treatments, and especially in the muddy water and over-heading submergence treatments. The content of soluble sugar in grains increased until 11 days after heading in the control and decreased after 11 days after heading. The soluble sugar content was temporarily decreased for the 24 h treatment in all submergence treatments, but no significant change was observed from the 24 h treatment to the 96 h treatment.

**Fig. 2:**
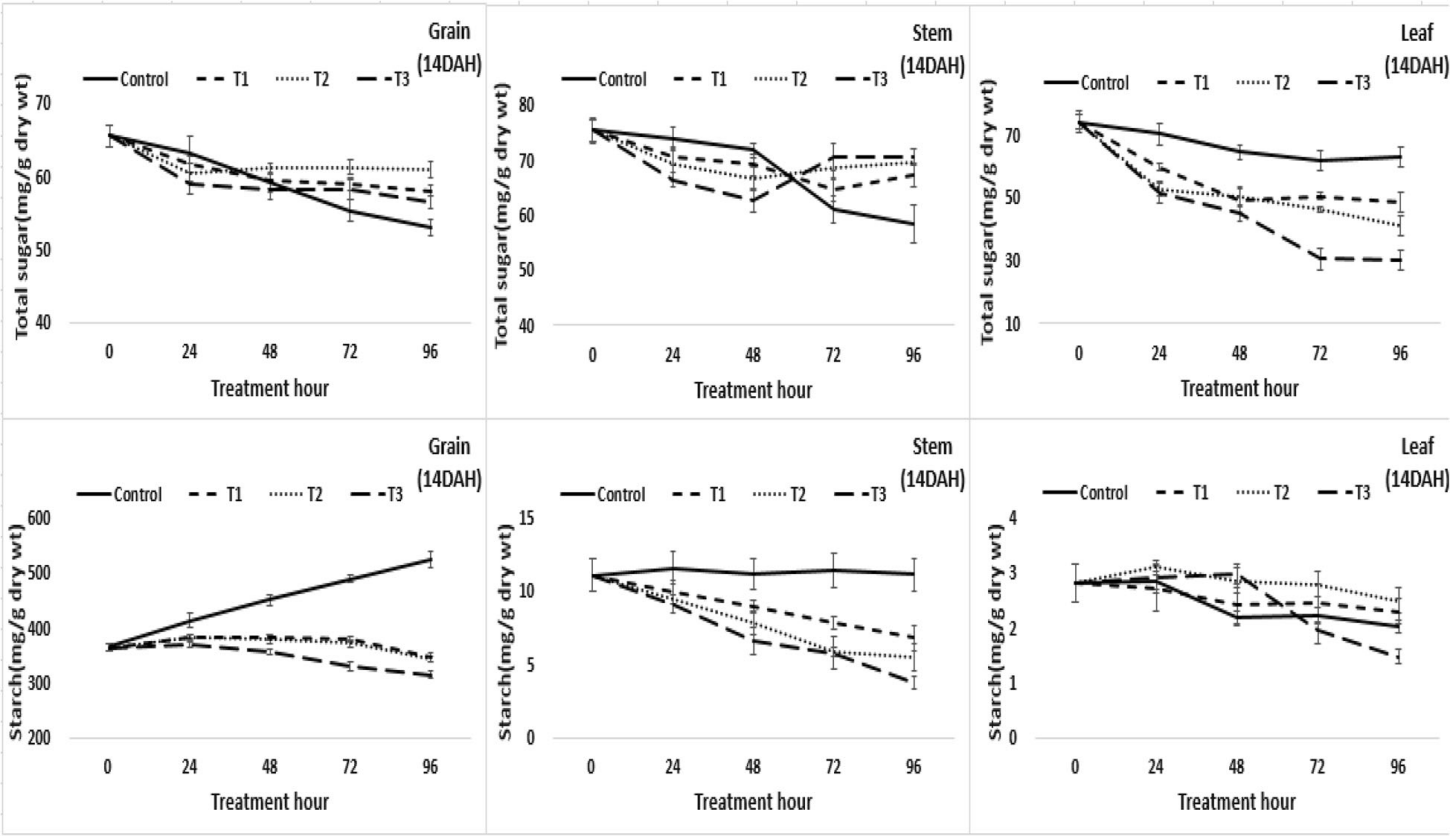
Changes in the soluble carbohydrate content. The changes in soluble carbohydrate content(total sugar and starch content) in grain, stems, and leaves affected by the flooding treatment hour at 14 days after heading(DAH). The treatment was carried out for up to 96 h and the samples were used in five replicates. T1: overheading and clear water flooding condition. T2: flag leaf exposure and muddy water flooding condition. T3: overheading and muddy water flooding condition

In stems, starch content decreased in all treatments, including the control, and decreased more rapidly in the submergence treatments compared to that of the control. However, soluble sugar content tended to increase somewhat in contrast to the decrease in starch content in the submergence treatments. For leaves, the starch content tended to decrease in the muddy water and over-heading submergence treatments after 48 h, but there was no significant increase or decrease in other treatments. The soluble sugar content decreased in all treatments, and was more severe in the submergence treatments than the control. In grains, the starch content increased in the control at 7 days after heading, but the starch content decreased in the muddy water and over-heading submergence treatment plots after 24 h and the starch content decreased in the muddy water and leaf exposure and clear water and over-heading submergence treatment plots after 48 h. The decrease of starch content in grain tended to be somewhat larger at 14 days after heading than that of 7 days after heading (Fig. 2). These results were thought to occur because the amount of carbohydrate stored in stems and leaf sheaths before heading was mostly exhausted by 14 days after heading, and eventually the movement of stored carbohydrates from stems and leaf sheaths to grain was slightly smaller than that of 7 days after heading. The response in leaves was similar to that of 7 days after heading.

**Fig. 3:**
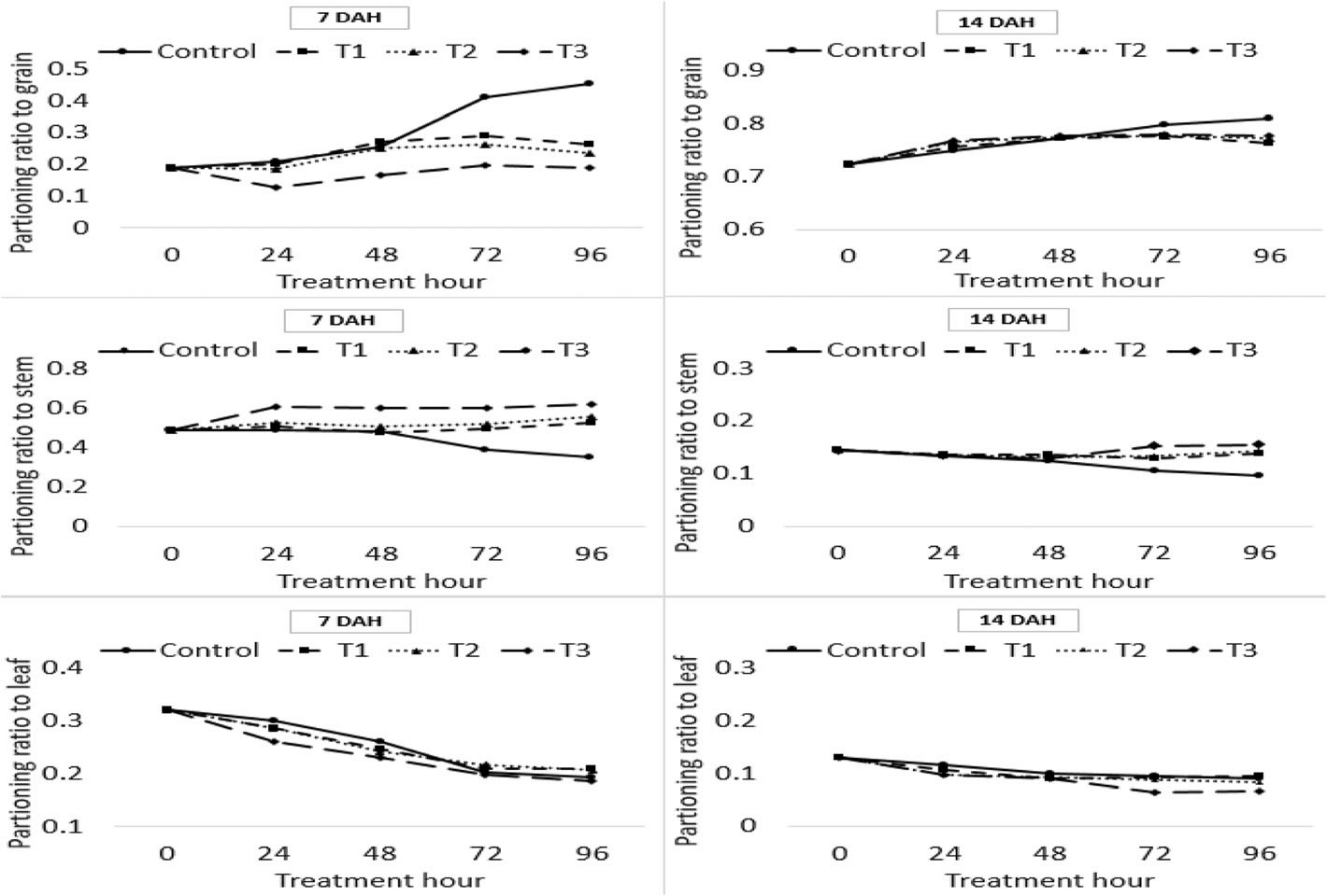
Changes of distribution ratio of organs. The changes in the distribution ratio for organs (grain, stem, leaf) was affected by flooding treatment hour at 7, 14 days after heading(DAH). The treatment was carried out for up to 96 h and the samples were used in five replicates. T1: overheading and clear water flooding condition. T2: flag leaf exposure and muddy water flooding condition. T3: overheading and muddy water flooding condition

The changes in the partitioning ratio for organs (grain, stem, leaf) was affected by flooding treatment during the ripening stage as shown in Fig. 3. Although the distribution of assimilation products to grain increased, and decreased to stems and leaves, according to the ripening progress in the control, in the submergence treatment plots the distribution of assimilation products to stems increased, resulting in an inhibited supply of assimilation products to grain.

### Differences in the relative expression of genes associated with sucrose and starch synthesis metabolism

**Fig. 4:**
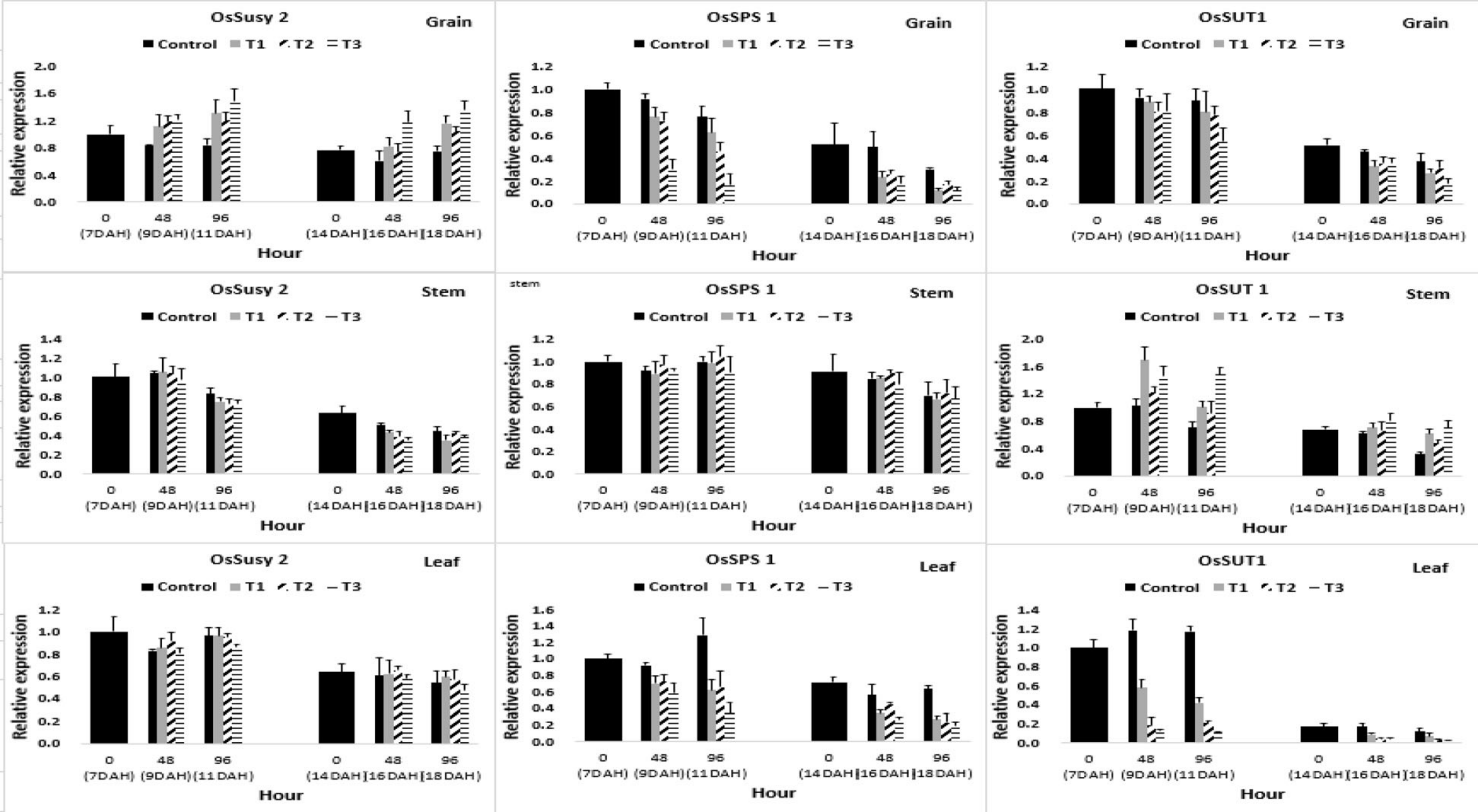
Difference in relative expression of genes associated with sucrose metabolism. Those genes affected by flooding treatment hour at 7, 14 days after heading(DAH). Susy2: sucrose synthase 2, SPS1: sucrose phosphate synthase1, SUT1: sucrose transporter. T1: overheading and clear water flooding condition. T2: flag leaf exposure and muddy water flooding condition. T3: overheading and muddy water flooding condition

The assimilation products synthesized by photosynthesis are converted into sucrose and are transported to the grain during ripening stage. The sucrose transported to the grain is converted to ADP-glucose, which is the final substrate for starch synthesis, via UDP-glucose, glucose-1-phosphate, and glucose-6-phosphate, and ADP-glucose is used in starch synthesis [29, 30]. The relative expression of the genes involved in sucrose metabolism, which was affected by submergence in the ripening stage, is shown in Fig. 4. The expression of sucrose synthase (OsSusy), which converts sucrose to UDP-glucose and fructose [31, 32], increased in grains and leaves, but not in stems. The expression of sucrose phosphate synthase (OsSPS), which converts UDP-glucose to sucrose [33, 34], tended to decrease in grains and leaves, but not in stems. In addition, the expression level of the sucrose transporter (OsSUT), which regulates sucrose transport [35], decreased in the grains and leaves, but increased somewhat in the stems. In summary, the expression of OsSPS associated with sucrose accumulation [36] decreased in grains and leaves, but OsSUT expression increased in the stems. These expression characteristics of the sucrose-related genes were consistent with the results in Fig. 3, where the assimilation products were concentrated in the stems and not in the grains and leaves.

**Fig. 5:**
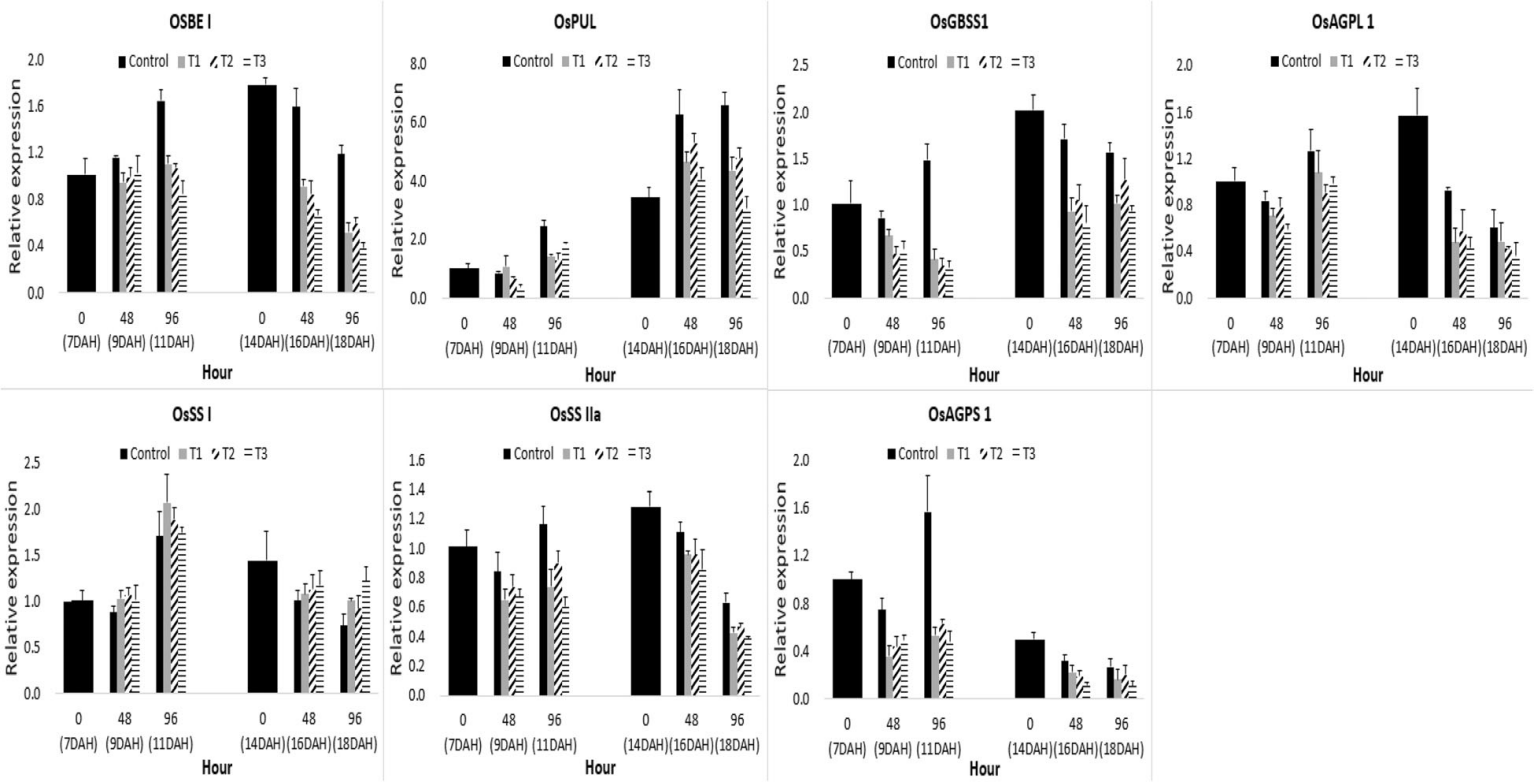
Difference in relative expression of genes associated with starch synthesis. Those genes affected by flooding treatment hour at 7, 14 days after heading(DAH). SBE1: starch branching enzyme1, PUL: debranching enzyme, GBSS1: granule-bound starch synthase1, AGPL: ADP-glucose pyrophosphorylase, SS: sucrose synthase, T1: overheading and clear water flooding condition. T2: flag leaf exposure and muddy water flooding condition. T3: overheading and muddy water flooding condition

ADP-glucose, which is the final substrate for starch synthesis, is converted from glucose-1-phosphate by the action of ADP-glucose pyrophosphorylase [37, 38]. Then, ADP-glucose is synthesized as amylopectin and amylose by soluble starch synthase and granule-bound starch synthase (OsGBSS) to form starch [39, 40]. In addition, amylopectin is regulated in the structure and chain length by the influence of the starch branching enzyme (OsSBE) and debranching enzyme (OsPUL) in addition to starch synthase [41]. The relative expression of the genes involved in starch synthesis, which is affected by submergence in the ripening stage, is shown in Fig. 5. The expression level of sucrose synthase I (OsSS I) tended to increase somewhat in submergence treatments, but the expression levels of ADP-glucose pyrophosphorylase large unit (OsAGPL), ADP-glucose pyrophosphorylase small unit (OsAGPS), OsSBE, OsPUL, and OsGBSS tended to decrease. In particular, the expression levels of OsPUL, which regulate the structure of amylopectin and length of the glycosidic bond chain, was significantly increased in submergence treatments at 14 days after heading compared to that of 7 days after heading.

### Sequencing and selection of differentially expressed genes

The results of the clean reads, which were obtained from the rice grain, stem, and leaf organs by trimming the short reads that were sequenced with the Illumina Hiseq platform, are shown in Table 1. The total number of reads ranged from at least 11,032,891 to a maximum of 17,673,004. The total length of the reads ranged from at least 945,089,151 to a maximum of 1,606,463,841, and the average length of the reads ranged from a minimum of 85.40 to a maximum of 90.90. The mapping results using these clean reads are shown in Table 2. Because the total number of reads between samples varied by 1.1 to 1.17 times, reads were randomly extracted to minimize errors during read counts and normalization, and the total number of reads was 11,032,891 based on the smallest number of seed control treatments. The mapping rate was 83 to 87%, with an average of 84.98%.

**Table 1:** Trimmed sequencing data

Sample description		Num. of clean reads	Avg. length	Total length (bp)
Seed	Control	11,032,891	85.7 ± 2.2	945,089,151 ± 24,735,160
	Submergence	12,595,149	85.4 ± 3.0	1,075,509,880 ± 38,212,576
Stem	Control	12,765,878	87.7 ± 3.3	1,119,579,740 ± 42,307,259
	Submergence	12,654,727	88.0 ± 1.7	1,113,925,917 ± 22,110,523
Leaf	Control	16,376,497	90.8 ± 2.8	1,486,987,894 ± 46,242,526
	Submergence	17,673,004	90.9 ± 2.8	1,606,463,841 ± 49,099,815

The results of the clean reads, which were obtained from the rice grain, stem, and leaf organs by trimming the short reads that were sequenced with the Illumina Hiseq platform. RNA was extracted from the samples (seed, stem, and leaf), which were treated with muddy water-irrigation for 4 days on the 14th day after heading, and sequencing was performed on the extracted RNA, which was the same RNA sample used for real-time PCR with three replicates

**Table 2:** Statistics of read mapping to reference gene set

Sample description		Total cleaned reads (ea)	Aligned 0 times		Aligned exactly 1 time		Aligned ≥1 times		Mapping rate	
			Reads (ea)	%	Reads (ea)	%	Reads (ea)	%	Reads (ea)	%
Seed	control	11,032,891	1,399,412 ± 8197	13	4,775,660 ± 1047	43	4,857,820 ± 7150	44	9,633,480	87
	submergence	11,032,891	1,622,211 ± 2966	15	4,797,957 ± 1901	43	4,612,724 ± 4867	42	9,410,680	85
Stem	control	11,032,891	1,738,721 ± 780	16	4,665,276 ± 1318	42	4,628,895 ± 2098	42	9,294,171	84
	submergence	11,032,891	1,917,617 ± 6293	17	4,463,265 ± 1225	40	4,652,010 ± 5068	42	9,115,274	83
Leaf	control	11,032,891	1,471,592 ± 6163	13	4,785,651 ± 3852	43	4,775,648 ± 2311	43	9,561,299	87
	submergence	11,032,891	1,790,521 ± 6763	16	4,385,443 ± 9853	40	4,852,428 ± 3275	44	9,242,371	84

Because the total number of reads between samples varied by 1.1 to 1.17 times, reads were randomly extracted to minimize errors during read counts and normalization, and the total number of reads was 11,032,891 based on the smallest number of seed control treatments. The mapping rate was 83 to 87%, with an average of 84.98%

**Fig. 6:**
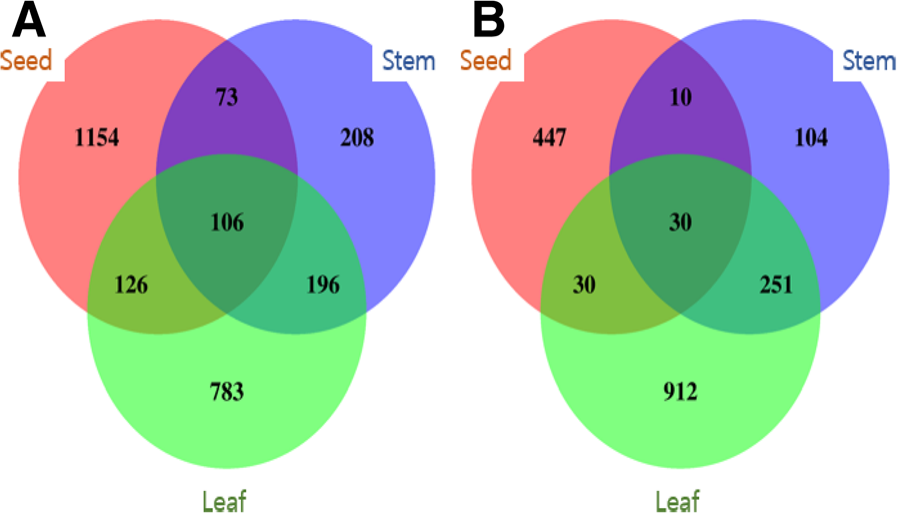
Venn-diagram of differentially expressed genes. The number of genes obtained by summarizing the overlapping parts of each organ (grain, stem, and leaf) using selected useful genes (DEGs) affected by flooding treatment at 14 days after heading. **a**: Up-regulated, **b**: Down-regulated

The results of selecting the differentially expressed genes (DEGs) according to submergence effects on the grain, stem, and leaf organs are shown in Additional file 1: Figure S1. As shown in the figure, the number of up-regulated DEGs was 1459 in grain, 583 in stems, and 1211 in leaves. The highest number of up-regulated DEGs was found in grain, being 1459. The number of down-regulated DEGs was 517 in grain, 395 in stems, and 1223 in leaves. The highest number of up-regulated DEGs was found in the leaves, being 1223. The information on the gene id, fold change value for selected DEGs, gene ontology (GO), and KEGG analysis results are shown in Additional file 2: Table S3. The MA-plots of differentially expressed genes (DEGs) between the control and submergence treatment for each tissue are shown in Additional file 3: Figure S2. In addition, the results obtained by summarizing the overlapping parts of each organ (grain, stem, and leaf) using selected useful genes (DEGs) is shown in Fig. 6. The number of DEGs that were up-regulated and did not overlap with each organ were the most abundant in grain, being 1154, and least in the stem, being 208 genes. In addition, among the up-regulated genes, the number of overlapping genes was the highest at 196 in the stems and leaves, the least, at 73, in the grain and stems. Conversely, the number of DEGs that were down-regulated and did not overlap with each organ were the most abundant in leaves with 912, and least in the stem, with 104 genes. Furthermore, the number of the DEGs that were up-regulated and overlapped with that of another organ was the most abundant between the stem and leaf with 196, and least between the grain and stem, with 73 genes. Conversely, the number of DEGs that were down-regulated and overlapped with that of another organ were the most abundant between the stem and leaf with 251, and least between the grain and stems, with 10 genes. Information on the genes represented by the Venn-diagram is shown in Additional file 4: Table S4.

### Gene ontology (GO) analysis

**Fig. 7:**
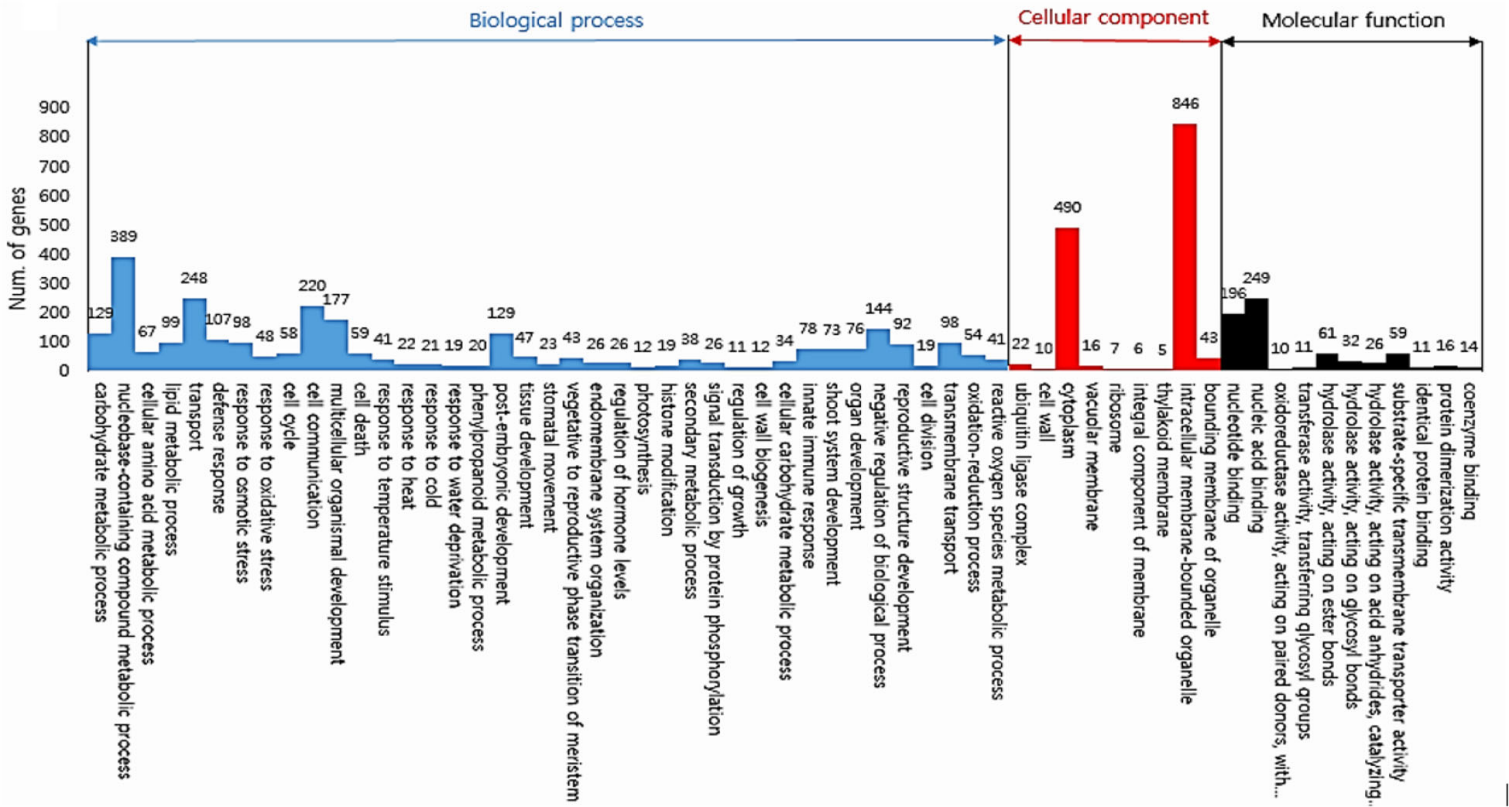
Gene ontology enrichment analyses of datasets obtained by RNA-Seq. up-regulated genes in seed. According to biological processes, a high proportion of up-regulated DEGs mapped to “biological process” and “cellular process”. According to cellular components, a high proportion of the up-regulated DEGs mapped to “cytoplasm” and “intracellular membrane-bounded organelle”. According to molecular functions, the a high proportion of up-regulated DEGs mapped to “nucleic acid binding” and “nucleotide binding” under the submergence treatments

To understand the functions of all these DEGs, we mapped them to terms in the GO database, searching for significantly enriched GO terms compared to the reference gene background (Fig. 7, Additional file 5: Figure S3).

In grain, firstly, according to biological processes, a high proportion of up-regulated DEGs mapped to “biological process” and “cellular process”, and a high proportion of the down-regulated DEGs mapped to “transport” and “carbohydrate metabolic process” under the submergence treatments. Secondly, according to cellular components, a high proportion of the up- and down-regulated DEGs mapped to “cytoplasm” and “intracellular membrane-bounded organelle” under the submergence treatments. Finally, according to molecular functions, a high proportion of up-regulated DEGs mapped to “nucleic acid binding” and “nucleotide binding” and that of down-regulated DEGs mapped to “cation binding” and “nucleic acid binding” under the submergence treatments.

In the stem, firstly, according to biological processes, a high proportion of the up-regulated DEGs mapped to “transport,” “multicellular organismal development,” and “carbohydrate metabolic process” and a high proportion of the down-regulated DEGs mapped to “transport” and “phosphorus metabolic process” under the submergence treatments. Secondly, according to cellular components, a high proportion of the up-regulated DEGs mapped to “cytoplasm” and “thylakoid” and a high proportion of the down-regulated DEGs mapped to “cytoplasm” and “intracellular membrane-bounded organelle” under the submergence treatments. Finally, according to molecular function, a high proportion of the up-regulated DEGs mapped to “nucleic acid binding” and “nucleotide binding” and a high proportion of the down-regulated DEGs mapped to “nucleotide binding” and “peptidase activity” under the submergence treatments.

In the leaf, firstly, according to biological processes, a high proportion of the up-regulated DEGs mapped to “transport,” “cell communication,” and “response to hormone” and a high proportion of the down-regulated DEGs mapped to “transport” and “phosphorus metabolic process” under the submergence treatments. Secondly, according to cellular components, a high proportion of the up- and down-regulated DEGs mapped to “cytoplasm” and “intracellular membrane-bounded organelle” under the submergence treatments. Finally, according to molecular function, a high proportion of the up-regulated DEGs mapped to “transferase activity” and “signaling receptor activity” and a high proportion of the down-regulated DEGs mapped to “cation binding” and “nucleotide binding” under the submergence treatments.

In summary, according to biological processes, the DEGs that mapped to “transport” and “carbohydrate metabolic process” constituted a high proportion, and according to cellular components, the DEGs that mapped to “cytoplasm” and “intracellular membrane-bounded organelle” constituted a high proportion, and according to molecular function, the DEGs that mapped to “cation binding” and “nucleic acid binding” constituted a high proportion in all organs under the submergence treatments.

### Clustering analysis

**Fig. 8:**
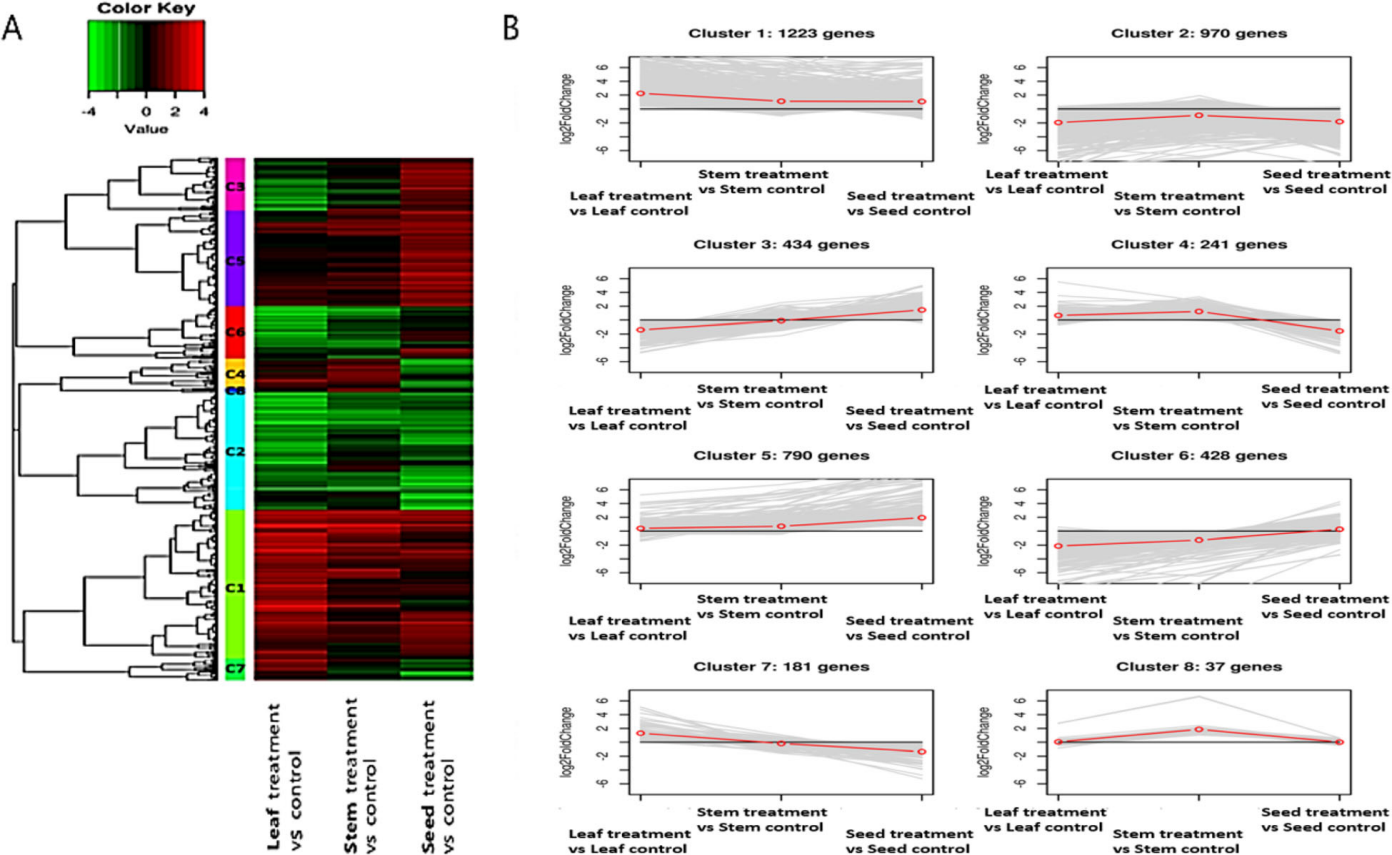
Hierarchical clustering analysis of differentially expressed genes. The DEGs with the same expression pattern were grouped into eight clusters from C1 to C8. **a**: Heat map, **b**: Line plot

The results of hierarchical clustering analysis of DEGs affected by flooding treatment during the ripening stage are shown in Fig. 8. The DEGs with the same expression pattern were grouped into eight clusters from C1 to C8, and the DEG information and the results of Gene Ontology (GO) and KEGG analysis for each cluster group are shown in Additional file 6: Table S5). Most biological processes, such as carbohydrate metabolic process, transport, response to oxidative stress, photosynthesis, and response to hypoxia were distributed from cluster 1 to cluster 8 and showed a uniform distribution pattern; however, in the case of phosphorus metabolic processes (GO: 0006793) related to nucleic acid, carbohydrates, and ATP metabolism, 216 DEGs were distributed only in cluster 2, which exhibited a down-regulation pattern of DEGs in all organs including grain, stems, and leaves, and these DEGs were mostly associated with carbohydrate metabolism. In addition, in the case of the responses to hormones (GO: 0009725), 239 DEGs were distributed only in cluster 1, which exhibited an up-regulation pattern of DEGs in all organs including grain, stems, and leaves, and these DEGs were mostly associated with hormones, such as auxin, gibberellin, and ethylene related to the avoidance strategy of submergence.

### Carbon fixation, photosynthetic electron transport, glycolysis, starch, and sucrose metabolism (KEGG pathway)

In the submergence condition, provision of oxygen becomes limited and anaerobic respiration occurs. Consequently, photosynthesis and assimilation production are expected to decrease because of light shading. The metabolism of each previously selected DEG was analyzed using the KEGG mapper to examine responses in metabolism, including photosynthesis, respiration, assimilation production, and starch synthesis in the submergence condition. Additional file 7: Figure S4 shows the results of the photosynthetic carbon fixation pathway analysis using the KEGG mapper based on submergence treatments. In the metabolism of the Calvin-Benson cycle, the genes that encode trans-ketolase (this enzyme converts D-fructose 6-phoshpate to erythrose-4-phosphate, and glyceraldehyde-3phosphate to riblose-5phosphate) and ribulose-phosphate 3-epimerase (this enzyme converts xylulose5-phosphate to rublose-5phosphate) based on the submergence treatment did not show any difference before and after the treatment. Only DEGs that encoded glyceraldehyde 3-phosphate dehydrogenase (GAPDH), which converts 1,3-bisphospho glycerate to glyceraldehyde-3phoshate, were up-regulated, and all the other DEGs associated with the Calvin Benson cycle were down-regulated (Additional file 8: Table S6). These results were similar to the findings of Yang et al. (1993) [42] where the expression of genes that encode GAPDH increased during environmental stress conditions, including heat shock, anaerobiosis, and increased sucrose supply treatments.

Additional file 9: Figure S5 demonstrates the analysis of the resulting photosynthetic electron transport pathway based on the flooding treatment. In the photosynthetic KEGG pathway, the majority was down-regulated out of the total of 55 processes of photosynthesis-associated metabolism annotated to the reference japonica. Among the metabolism processes that included DEGs that showed a difference of at least two-fold according to the irrigation condition, only seven demonstrated a reaction. Among the seven metabolism processes, photosystem II oxygen-evolving enhancer protein 3 (Psb Q) in was the only one that was up-regulated. Photosystem II 10 kDa protein (Psb R), photosystem II 22 kDa protein (Psb S), Cytochrome b6/f complex iron-sulfur subunit (Pet C), ferredoxin--NADP+ reductase (pet H), and F-type H + −transporting ATPase subunit (gamma a, b) were down-regulated. Photosynthetic electron transport pathway-associated DEGs according to the submergence treatment were identified, including LOC_Os07g37030, LOC_Os08g10020, and LOC_Os04g55960 (Additional file 10: Table S7).

The results of the metabolism pathway analysis according to the submergence treatments are shown in Additional file 11: Figure S6. Genes that encoded phosphoglucomutase, which converts α-D-glucose-1P to α-D-glucose-6P, were down-regulated in leaves, but the expression showed no difference in stems and grain. The processes in which α-D-glucose-6P was converted to phosphoenolpyruvate through glyceraldehyde-3P and glycerate-3P were all up-regulated both in stems and grain. The processes did not show any regular tendency in leaves, such as up- or down-regulation. The genes that encode pyruvate decarboxylase (4.1.1.1) and alcohol dehydrogenase (1.1.1.1) (which are associated with pyruvate’s ethanol formation through acetaldehyde) were up-regulated in all three organs: grain, stems, and leaves (Additional file 12: Table S8).

**Fig. 9:**
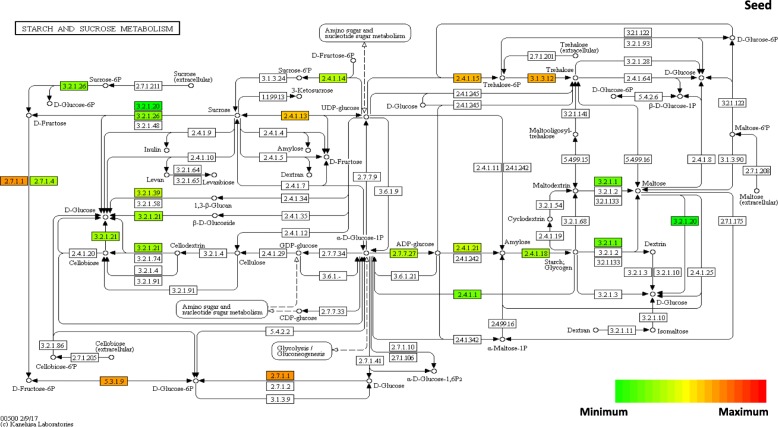
Changes in the reaction related to starch and sucrose metabolism in seed. The metabolism of each previously selected DEG was analyzed using the KEGG mapper

Figure 9 and Additional file 13: Figure S7 shows changes in sucrose and starch metabolism in the submergence conditions. Principal changes were observed in the process of synthesis from sucrose to starch, and those synthesizing beta-glucosidase and trehalose activity increased according to environmental stress. First, in the synthesis process of sucrose to starch in grain, the conversion of sucrose to D-fructose and to D-glucose (3.2.1.26) was down-regulated in all organs. Later, in the process of conversion to ADP-glucose, the synthesis substrate was α-D-glucose-1P through D-fructose-6P and D-glucose-6P was up-regulated in grain and stem, but no reaction was observed in leaves. Conversely, the process of sucrose conversion to UDP-glucose was up-regulated in grain, stems, and leaves, whereas ADP-glucose pyrophosphorylase, starch synthase, and starch branching enzyme were down-regulated in grain and leaves from the ADP-glucose synthesis (2.7.7.27) to starch synthesis (2.4.1.21, 2.4.1.18). In stems, only ADP-glucose pyrophosphorylase was up-regulated. In starch and sucrose metabolism, beta-glucosidase is associated with producing D-glucose in 1,3-βglucan, β-D-glucose, and cellulose (3.2.1.39, 3.2.1.21, 3.2.1.4). This beta-glucosidase has various functions, including producing defense-associated bioactive compounds during environmental stress, degradation of endosperm cell walls during germination, activation of phytohormones, and lignification, which produces lignin in cell walls and mesoderm [43]. The expression of genes encoding these functions was down-regulated in grain and leaves, and up-regulated in stems and associated DEGs (Additional file 14: Table S9) LOC-Os11g45710 (beta-glucosidase-like SFR2, chloroplastic), and LOC_Os01g71860 (glycosyl hydrolases family 17) were observed. Beta-glucosidase is also reported as a substance associated with the expression of genes induced by environmental stress, such as drought and salt [44]. Synthesis of trehalose, which is known to control starch synthesis by activating ADP-glucose pyrophosphorylase (AGPase), is controlled by trehalose 6-phosphate synthase (2.4.1.15), which produces trehalose-6phosphate in UDP-glucose, and by trehalose 6-phosphate phosphatase (3.1.3.12), which produces trehalose in trehalose-6phosphate. Similar to the experiment by Koble et al. (2005) [45] in which AGPase activity increased because of the increased expression of trehalose 6-phosphate phosphatase (TPP) gene, the expression of TPP and AGPase in leaves was down-regulated. Conversely, in grain the expression of AGPase was down-regulated despite the up-regulation of TPP expression, being different than that of leaves. The information of DEGs associated with different amounts of expression in each pathway is demonstrated in Additional files 8, 10, 12 and 14: Tables S6, S7, S8, and S9.

## Discussion

In rainfed lowlands vulnerable to flood in Southeast Asia, and West Africa, precipitation occurs in meter units, and a long-term precipitation occurs at times lasting for more than a month [46]. The International Rice Research Institute (IRRI) conducted numerous studies of SUB1A gene [47], which controls the survival properties during long-term flood stress by controlling the consumption of carbohydrate storage in anaerobic conditions, with the goal of developing flood-resistant rice that survives long-term raining periods. The institute developed a new rice variety by introducing SUB1A gene to rice, which does not contain the SUB1A gene. A back cross study was conducted to recover several traits, including head-rice ratio, and protein and amylose content [48, 49].

Recently, research has attempted to develop a breeding program for new flood-resistant varieties. Studies were conducted to identify a variety of genes and other QTLs that could have new effects in combination with the flood-resistant traits of SUB1, in addition to those of the QTL already associated with SUB1 that controls flood-resistant traits. However, little has been found in physiological and molecular biological terms [20, 50]. In Sharma’s study [21], to provide insights in to novel aspects of SUB1A-mediated tolerance, some intermediate tolerance varieties, such as IR64, carry the allelic variant SUB1A-2 and its derived tolerant near the isogenic line IR64-Sub1, which carries the SUB1A-1 allele were used to compared the expression of nearly 2500 rice TF genes. This identified distinct sets of transcription factor genes affected by submergence. Additionally, in Xiang’s study [22], to provide new insight into the mechanism of phytohormone-regulated submergence response in rice, paclobutrazol, which could significantly enhance rice seedling survival in submergence conditions, by retaining a higher level of chlorophyll content and alcohol dehydrogenase activity and decelerating the consumption of non-structure carbohydrate, was treated with rice. In total, 3936 differentially expressed genes (DEGs) that could enhance the submergence tolerance were identified by transcriptomic analysis. Among those DEGs, they selected CIPK15, MPK3, SD-1, and OScyt-inv1 as the key genes that are expected to play important roles in enhancing submergence tolerance. Moreover, in Hsu’s study [23], they conducted RNA-seq analysis in order to identify transcriptome characteristics for the difference in early growth between submergence tolerant and sensitive varieties during the germination stage under submergence conditions. A comparative analysis of biological metabolic pathways, including hormone synthesis, cell wall growth, glycolysis for six genotypes were conducted. In addition, candidate DEGs such as OsTPP7, HXK7, and PGM, which could affect early growth under submergence conditions, were selected. Similar to these studies, transcriptome analysis studies related to submergence stress have been conducted recently.

Reduced head-rice ratio and ripening rate following flood damage during the ripening period are likely caused by an excessive consumption of respiratory substrate caused by reduced photosynthesis and anaerobic respiration, and limited assimilation product supply to grain because of an increased stem distribution. In the submergence treatment plots the distribution of assimilation products to the stems increased, resulting in an inhibited supply of assimilation products to grain (Fig. 3). The supply of these assimilation products to the stems appears to occur for stem elongation, according to the avoidance strategy. These results are similar to those of Das [7], in which there was an increased carbohydrate content in the stems for elongation in the submergence treatment. This phenomenon was verified by the fact that the expression of sucrose phosphate synthase (SPS) associated in sink activity, which induced sucrose accumulation in tissues, was reduced in grain and leaves compared to that of the control, whereas the expression was maintained in stems. It was also demonstrated through the reduction of sucrose transporter (SUT) associated with sucrose transport in grain and leaves, whereas it increased in stems (Fig. 4). These results are similar to those of Adak [51] in that sucrose phosphate synthase activity decreased in submergence treatment in rice.

The limited supply of assimilation products to grain affected the starch synthesis metabolism. There was no difference in expression during the conversion from UDP-glucose synthesized from sucrose to α-D-glucose-1P. The conversion of α-D-glucose-1P to the final substrate of starch synthesis ADP-glucose, and the synthesis of starch synthase (SS) and starch branching enzyme (SBE) to amylose and amylopectin were all down-regulated (Fig. 9, Additional file 13: Figure S7). This was probably because part of UDP-glucose, which should be used for conversion to the ADP-glucose synthesis substrate α-D-glucose-1P, was synthesized to trehalose-6P and then to trehalose, as shown in the resulting increased expression of genes that encode trehalsoe-6P-sytnahse and trehalose 6-phosphate phosphatase (Fig. 9, Additional file 13: Figure S7). In Locke’s study [52], the involvement of low energy sensing during submergence was supported by dynamics in trehalose-6-phosphate and mRNAs encoding key enzymes, which were modulated by SUB1A. The role of trehalose synthesis in a condition with limited transport of assimilation product to grain should be investigated further. Meanwhile, the expression levels of OsPUL, which regulate the structure of amylopectin and length of the glycosidic bond chain, was significantly increased in submergence treatments at 14 days after heading compared to that at 7 days after heading(Fig. 5). These results suggest that the decrease in the expression of OsPUL in submergence treatments may have affected the results showing an significant increase in chalky grain [53] at 14 days after heading compared to that of 7 days after heading [54].

Pyruvate produced through the mean process was converted to acetyl-CoA in general aerobic respiration conditions, and produced energy, such as ATP through the TCA cycle. However, respiration becomes inefficient producing a much smaller amount of energy in anaerobic respiration because of the submergence compared to aerobic respiration. Alcohol fermentation or lactic acid fermentation generating ethanol, and lactate occurs in anaerobic respiration. The glycolysis/gluconeogenesis pathway affected by submergence treatment (Additional file 11: Figure S6) was analyzed. The conversion of glucose to pyruvic acid in the submergence treatments was up-regulated throughout the entire process in grain and stems. The amount of expression was greater in grain than stems. Conversely, both up- and down-regulations were observed, but no regular tendency was observed in the entire process in leaves. With respect to the alcoholic fermentation of anaerobic respiration in pyruvate that produces ethanol, the expression of DEGs, such as alcohol-dehydrogenase (LOC_Os11g10480, LOC_Os11g10510), aldehyde-dehydrogenase (LOC_Os02g49720), and pyruvate decarboxylase (LOC_11g38910) tended to all be up-regulated in grain, stems, and leaves. The difference in alcohol fermentation-associated DEG expression was substantially greater in grain (Additional file 12: Table S8). Phosphoenolpyruvate carboxykinase (LOC_Os10g13700, LOC_Os03g15050) (PCK) converts oxaloacetate (OAA) produced through the TCA cycle back to phosphoenolpyruvate. It was down-regulated in grain following the submergence treatment, but tended to be up-regulated in stems and leaves. PCK is known to decarboxylate OAA in C4 or CAM plant photosynthesis to provide CO_2_, or to control gluconeogenesis during germination or ripening [55]. However, little is known about its role in rice, and the PCK reaction in submergence treatment should be investigated further.

As it is known that gas diffusion slows down 10,000 times or more in submergence conditions relative to the atmosphere, and reduces photosynthesis [4], only DEGs that encoded GAPDH that converts 1,3-bisphospho glycerate to glyceraldehyde-3phoshate in the Calvin Benson cycle were up-regulated. Genes associated with the rest of the process were down-regulated. A study reported that GAPDH expression increased in response to environmental stresses, such as high temperature, drought, and hypoxia, in addition to its well-known role in glycolysis and the Calvin Benson cycle [42]. Kappachery et al. (2015) [56] examined the response of GAPDH-overexpressed transgenic potatoes in drought stress conditions. They observed that the survival rate increased in the drought stress condition compared to that of the control. This was probably because GAPDH played the role of distributing the carbohydrate supply to the pathways, including glycolysis and the Calvin Benson cycle, supplying additionally energy in the limited energy supply conditions because of the environmental stress [57]. Further study is required to elucidate its response and role in rice submergence conditions.

This study analyzed the changes observed in metabolism associated with the production of assimilation products, transportation to grain, and starch synthesis, which affects the ripening of rice in submergence conditions. The results established a network of changing responses between important factors in submergence conditions other than the resistance mechanism of SUB1A, and SK1 and 2 genes that previously were intensively studied. They also suggest important information regarding DEGs, and can be used as basic data for developing varieties and technology adaptive to domestic flood damage conditions. This will ultimately contribute to the stable production of rice produced in frequently flooded areas.

## Conclusions

The following are analysis results of the characteristics of assimilation product distribution and transcriptome according to rice ripening stages and damages in each flood condition.

1. In case of flood damage during the ripening period, the ratio of assimilation product distributed to stems is increased and that to grain decreased. The increased ratio of assimilation product distribution to stems is likely intended for extended aboveground growth according to the flood escape mechanism.
2. OsSPS is associated with sink activity, which induces the accumulation of sucrose in tissues. Regarding its distribution, it was observed in that the expression of OsSPS decreased in grain and leaves in submergence conditions compared to that of the control, and that of OsSUT associated with sucrose transport decreased in grain and leaves, whereas it increased in stems.
3. There was no difference in the expression of genes associated with the conversion of UDP-glucose produced from sucrose transported to grain to α-D-glucose-1P. Conversely, those genes were down-regulated that encoded SS and SBE associated with the conversion of α-D-glucose-1P to the final starch synthesis substrate ADP-glucose, and with the synthesis of amylose and amylopectin.
4. DEGs were selected that demonstrated significant differences in expression in each of organ, grain, stems, and leaves, according to flooding treatment. Expression patterns of the selected genes were analyzed with clustering analysis. The cluster group was categorized into C1 through C8.
5. The selected DEG metabolism processes associated with photosynthesis, respiration, assimilation production, and starch synthesis were analyzed using the KEGG mapper. Glyceraldehyde 3-phosphate dehydrogenase (GAPDH), trehalose 6-phosphate phosphatase (TPP), and phosphoenolpyruvate carboxykinase (PCK) demonstrated specific responses in the metabolism processes. Based on this, several DEGs were selected for further study of their role in flood damage.

## Methods

### Experiment materials and method

Nampyeongbyeo (rice of japonica type) was used in this experiment. Nampyeonbyeo was obtained from the Nation institute of crop science in Korea. Three seedlings were transplanted in 1/5000 a plots, and grown in natural weather conditions until the submergence treatment. Submergence treatment was conducted at the submergence treatment facilities based in the Artificial Weather Block of the National Institute of Crop Science. The average temperature of submergence treatment was 24 °C (minimum 19 °C and maximum 29 °C), and pH was set between 7 and 7.5. The treatment was divided into clear water and muddy water. The muddy water was conditioned using field dirt focusing on dissolved oxygen and shade level. Changes of the water condition by submergence treatment are shown in Additional file 15: Table S1. Dissolved oxygen was categorized into before flooding treatment (8.0 mg/L); 24 h after the flooding treatment (7.4 mg/L); 48 h after (6.6 mg/L); 72 h after (5.1 mg/L); and 96 h after (4.0 mg/L). Muddy water was categorized into the before flooding treatment (7.2 mg/L); 24 h after the flooding treatment (6.1 mg/L); 48 h after (4.5 mg/L); 72 h after (2.7 mg/L); and 96 h after (2.1 mg/L). To analyze the change of insolation by flooding treatment, the amount of insolation was measured at 11 am in the morning for each treatment condition. The insolation of clear and muddy waters was measured at underwater flag leaf level. Flood treatment was performed 7 and 14 days after heading according to each ripening stage in 59% shade to natural light for clear water, and 94% shade for muddy water. The treated plots were named T1: Overheading and clear water, T2: Flag leaf exposure and muddy water, and T3: Overheading and muddy water flooding. Names were based on each flooding condition.

### Content of starch and free sugar

Starch content was measured and analyzed using a starch assay kit (Sta20, Sigma, USA). A 25 mg ground sample (grain, leaf, and stem) was treated with 5 mL of 80% ethanol to eliminate maltodextrin and glucose. Then, 1 mL DMSO was added to the sample, and it was heated in boiling water for 5 min. Resistant starch was eliminated. Thermostable a-amylase and MOPS buffer were added to the sample, and it was kept in boiling water for 10 min. Amyloglucosidase was then added. The sample was kept at 50 °C for 30 min to decompose starch. Glucose content was measured at 540 nm wavelength by color reaction.

For free sugar analysis, 1 g of frozen sample (grain, flag leaf, stem) was treated with 20 mL of 50% acetonitrile solution at 30 °C, and stirred at 200 rpm for 18 h. The extracted sample was centrifuged at 4000 rpm for 10 min. The supernatant was removed, and centrifuged at 13500 rpm for 10 min. It was then filtered at 0.2 μm using a 1 mL syringe, and was injected to a UPLC vial. ACQUITY UPLC (Waters, USA) and ACQUITY UPLC BEH amide column (2.1 × 50 mm, 1.7 μm) were used for the analysis. Phase A was 80% ACN with 0.2% TEA, and Phase B 30% ACN with 0.2% TEA. Flow rate was 0.150 mL/min, and flow profile 93% / 7% B.

### RNA extraction and gene expression

For RNA extraction, each of the three samples (in the seed, stem, and leaf), which were treated with muddy water-irrigation for 4 days on the 14th day after heading, were taken from each ripening stage, and were immediately frozen using liquid nitrogen. The samples were stored at − 80 °C. Total RNA was extracted according to the Chang et al. (1993) protocol [58]. cDNA was synthesized using Primescript RT reagent kit with gDNA eraser (Takara bio Inc., Japan). In Realtime PCR, SYBRGreen (SYBR Realtime PCR Master Mix, Toyobo) was used for fluorescent dye. The analysis was conducted with three replicates per tissue using the Roter-Gene TM6000 (Corbett Research, Australia). The primer sequences are shown in Additional file 16: Table S2.

### Sequence pretreatment

RNA was extracted from the samples (seed, stem, and leaf), which was treated with muddy water-irrigation for 4 days on the 14th day after heading, and sequencing was performed on the extracted RNA, which was the same RNA sample used for real-time PCR with three replicates. For pretreatment of the sequenced short reads, duplicated reads produced by the PCR during the library development were filtered through in-house scripts. Final trimmed data were refined using Dynamic trim and Length sort of the SolexaQA [59] package. Dynamic trim renders data as refined cleaned reads by trimming bad quality base on both ends of short reads according to the Phred score. Length sort eliminates reads with too much base cut off by Dynamic trim. Phred score was > 20 in Dynamic trim, and short read length was > 25 bp in Length sort.

### Read mapping, normalization, annotation

Read mapping was conducted using Bowtie (v.2.1.0) software based on the Langmead and Salizberg (2012) [60] method (mismatch ≤2 bp, penalty). Expression was measured by the total number of reads mapped on each gene. The R package DEseq library [61] was implemented to calculate proper gene expression values for samples with data deviation. To examine the function of selected genes, the annotation information provided by Phytozome [62] DB was used.

### Selection of DEGs

For selection of DEGs in each sample, both the 2-fold change method, which verifies a difference in expression greater than twice between expression values mapped in each gene in each sample, and the binomial test method in which the adjusted *P*-value (FDR) satisfying 0.01 or lower were simultaneously applied [63]. In this study, it was defined as up-regulation if log_2 (Fold Change) was greater than 1, and down-regulation if smaller than − 1. And this value can be found in Additional file 2: Table S3.

### Analysis of DEGs function and expression patterns

Alignment was conducted using DEG candidates and sequences provided by GO DB for gene ontology analysis [64]. The total number of genes by function was set at counts > 1 for thresholds. GO depths were set at 3, and were categorized into three functional categories, BP (Biological Process), CC (Cellular Component), and MF (Molecular Function). Annotation (filter standard: e-value ≤1e-10, best hits) was conducted with amino acid sequence and BLASTX provided on KEGG for KEGG analysis. Clustering analysis was performed to identify gene expression patterns using selected DEG information. Hierarchical clustering analysis was conducted using R amap [65] and gplots library.

## Additional files

Additional file 1: **Figure S1.** The number of differentially expressed genes (DEGs) of organs. The results of selecting the DEGs according to 96 h submergence effects on the grain, stem, and leaf organs at 14 days after heading. (DOCX 253 kb)

Additional file 2: **Table S3.** The differentially expressed genes (DEGs) in seed, stem, and leaf response to submergence treatment for 94 h at 14 days after heading. (XLSX 1215 kb)

Additional file 3: **Figure S2.** MA-plots of differentially expressed genes (DEGs) between control and submergence treatment for each tissue. (DOCX 104 kb)

Additional file 4: **Table S4.** Venn-diagram of differentially expressed genes affected by flooding treatment during the ripening stage. (XLSX 513 kb)

Additional file 5: **Figure S3.** Gene ontology enrichment analyses of datasets obtained by RNA-Seq. (DOCX 495 kb)

Additional file 6: **Table S5.** The hierarchical clustering analysis data of the differentially expressed genes (DEGs) in seed, stem, and leaf responses to submergence treatment. (XLSX 722 kb)

Additional file 7: **Figure S4.** Changes in the reaction related to carbon fixation in photosynthetic organism metabolism in leaves. (DOCX 116 kb)

Additional file 8: **Table S6.** The KEGG pathway enrichment analysis of the differentially expressed genes (DEGs) related to carbon fixation in photosynthetic organism metabolism. (XLSX 30 kb)

Additional file 9: **Figure S5.** Changes in the reaction related to photosynthetic electron transport metabolism. (DOCX 206 kb)

Additional file 10: **Table S7.** The KEGG pathway enrichment analysis of the differentially expressed genes (DEGs) related to photosynthesis (electron transport and antenna proteins) metabolism. (XLSX 335 kb)

Additional file 11: **Figure S6.** Changes in the reaction related to glycolysis, gluconeogenesis metabolism in seed, stems, and leaves. (DOCX 312 kb)

Additional file 12: **Table S8.** The KEGG pathway enrichment analysis of the differentially expressed genes (DEGs) related to glycolysis/gluconeogenesis metabolism. (XLSX 36 kb)

Additional file 13: **Figure S7.** Changes in the reaction related to starch and sucrose metabolism in stems and leaves. (DOCX 277 kb)

Additional file 14: **Table S9.** The KEGG pathway enrichment analysis of the differentially expressed genes (DEGs) related to starch and sucrose metabolism. (XLSX 39 kb)

Additional file 15: **Table S1.** Changes of water condition by submergence treatment during ripening period. (DOCX 16 kb)

Additional file 16: **Table S2.** Gene specific PCR primer sets for quantitative RT-PCR amplification. (DOCX 17 kb)

## Abbreviations

DEG: Differentially expressed gene
GO: Gene ontology
Heat map: A graphical representation of data where the individual values contained in a matrix are represented as colors.
KEGG: Kyoto Encyclopedia of Genes and genomes
NSC: Nonstructural carbohydrate
PCR: Polymerase chain reaction
QTL: Quantitative trait locus
T1: Overheading and clear water flooding condition
T2: Flag leaf exposure and muddy water flooding condition
T3: Overheading and muddy water flooding condition
TCA Cycle: Tricarboxylic acid cycle
