# Supplementary material for: Analysis of the distribution of assimilation products and the characteristics of transcriptomes in rice by submergence during the ripening stage
Source: BMC Genomics. 2019 Jan 8;20:18. doi: 10.1186/s12864-018-5320-7 (PMC6323827; doi:10.1186/s12864-018-5320-7)
Supplement: Supplementary file 1 — Figure S1. The number of differentially expressed genes (DEGs) of organs. The results of selecting the DEGs according to 96 h submergence effects on the grain, stem, and leaf organs at 14 days after heading. (DOCX 253 kb) [file 12864_2018_5320_MOESM1_ESM.docx]

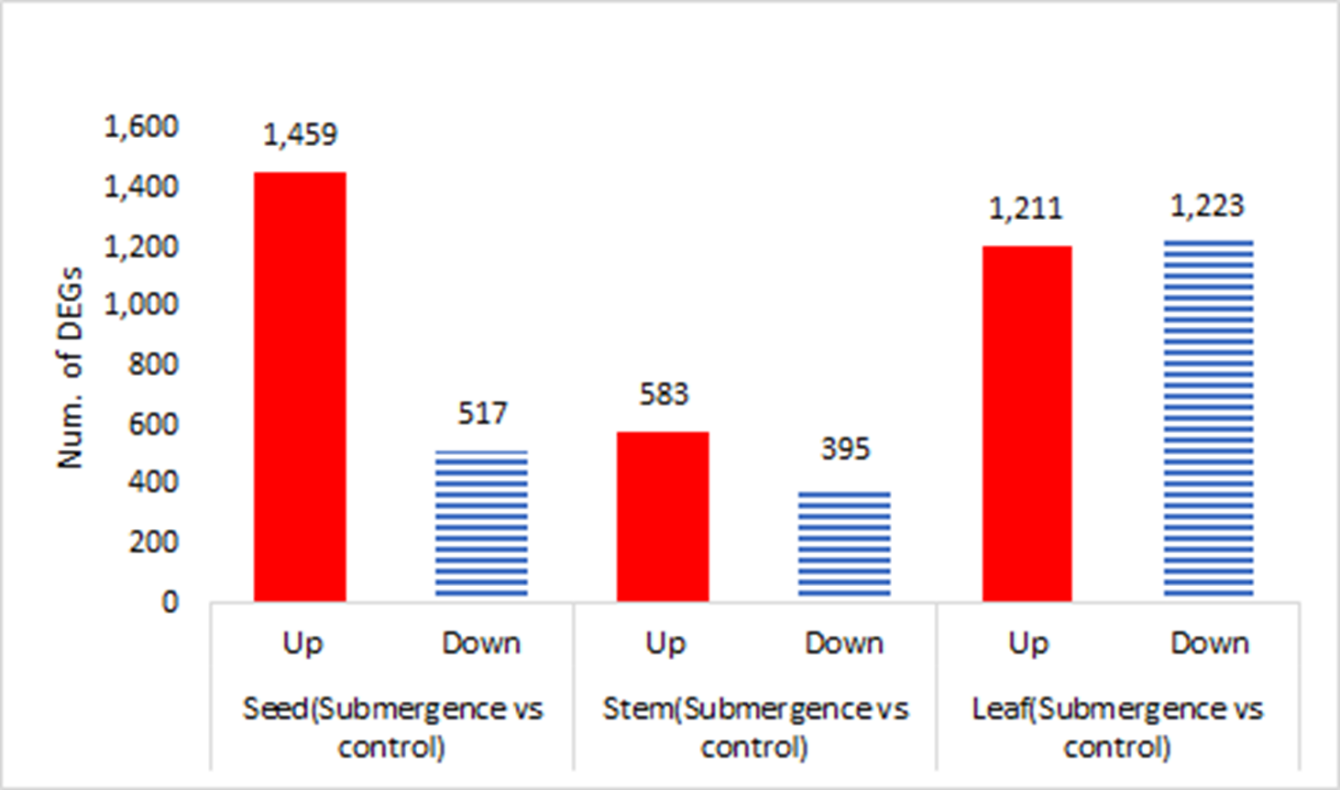


Figure S1. The number of differentially expressed genes (DEGs) of organs. The results of selecting the DEGs according to 96 hours submergence effects on the grain, stem, and leaf organs at 14 days after heading.
