# Supplementary material for: Analysis of the distribution of assimilation products and the characteristics of transcriptomes in rice by submergence during the ripening stage
Source: BMC Genomics. 2019 Jan 8;20:18. doi: 10.1186/s12864-018-5320-7 (PMC6323827; doi:10.1186/s12864-018-5320-7)
Supplement: Supplementary file 3 — Figure S2. MA-plots of differentially expressed genes (DEGs) between control and submergence treatment for each tissue. (DOCX 104 kb) [file 12864_2018_5320_MOESM3_ESM.docx]

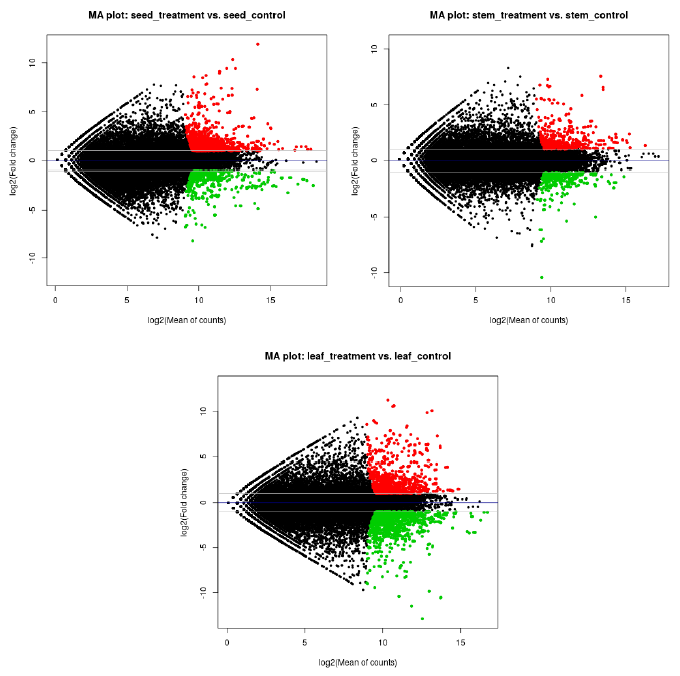


Figure S2. MA-plots of differentially expressed genes (DEGs) between control and submergence treatment for each tissue. The X-axis represents the mean expression value of the control and treatment on a log2 scale, and the Y-axis represents the fold change, a comparison between two samples, on a log2 scale.
