# Supplementary material for: Analysis of the distribution of assimilation products and the characteristics of transcriptomes in rice by submergence during the ripening stage
Source: BMC Genomics. 2019 Jan 8;20:18. doi: 10.1186/s12864-018-5320-7 (PMC6323827; doi:10.1186/s12864-018-5320-7)
Supplement: Supplementary file 5 — Figure S3. Gene ontology enrichment analyses of datasets obtained by RNA-Seq. (DOCX 495 kb) [file 12864_2018_5320_MOESM5_ESM.docx]

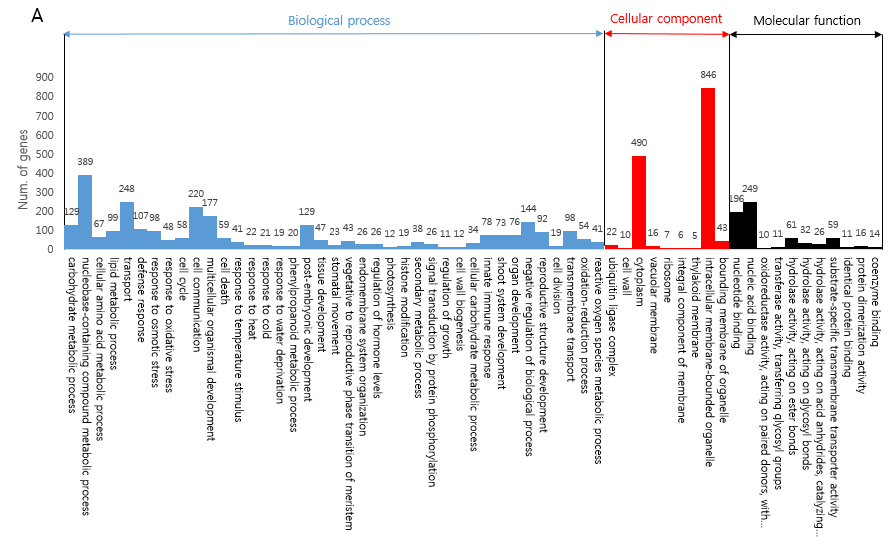

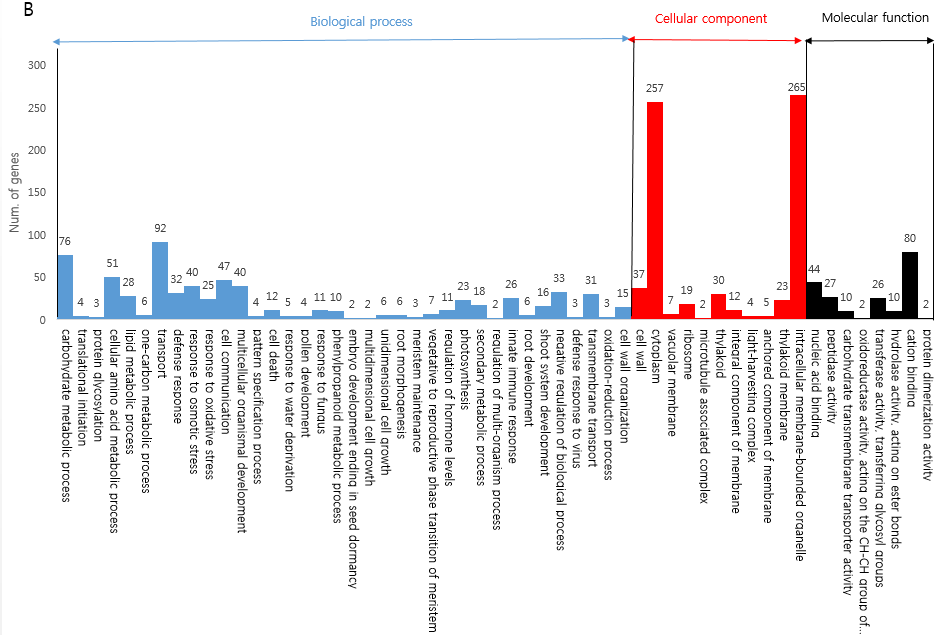


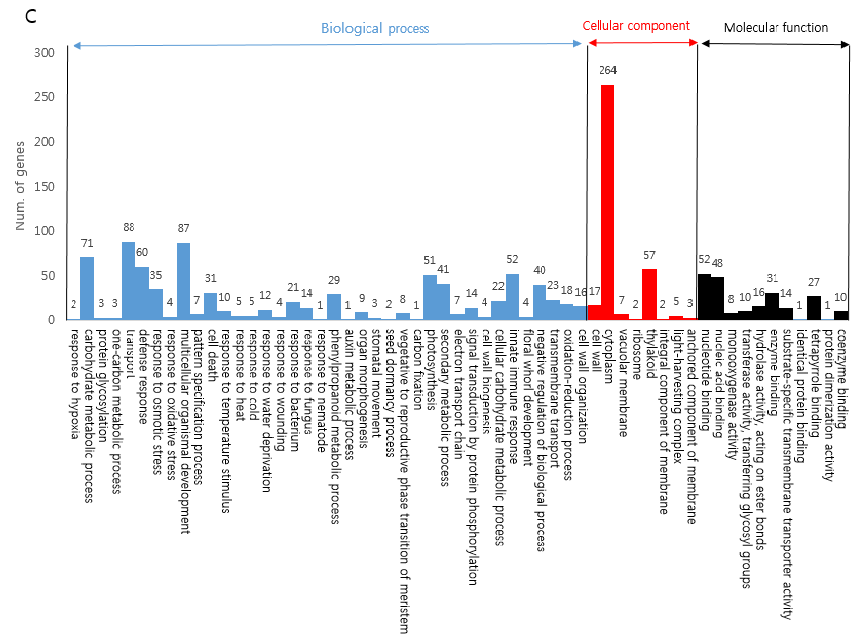


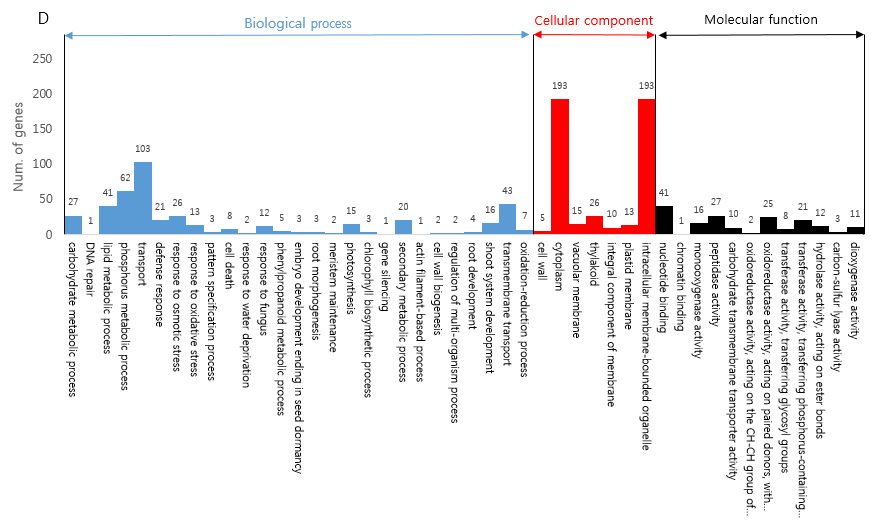


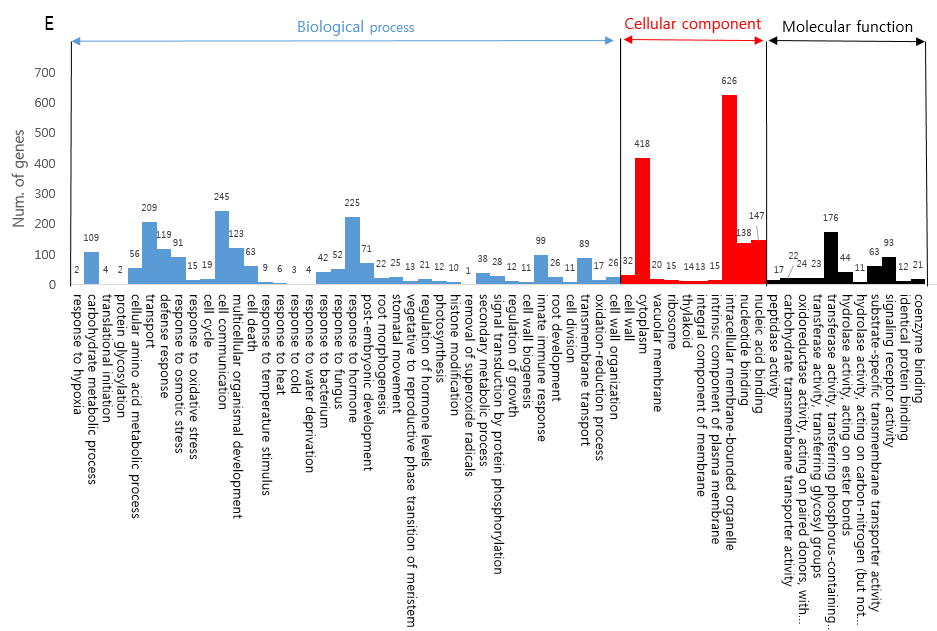


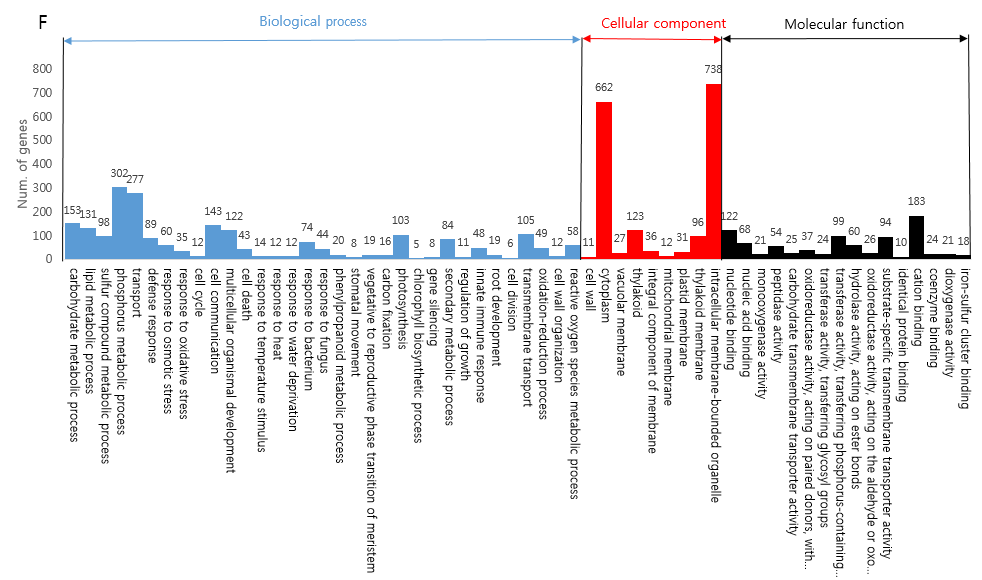


Figure S3. Gene ontology enrichment analyses of data sets obtained by RNA-Seq. A : up-regulated genes in seed B : down-regulated genes in seed C : up-regulated genes in stem D : down-regulated genes in stem E : up-regulated genes in leaf F : down-regulated genes in leaf
