# Supplementary material for: Analysis of the distribution of assimilation products and the characteristics of transcriptomes in rice by submergence during the ripening stage
Source: BMC Genomics. 2019 Jan 8;20:18. doi: 10.1186/s12864-018-5320-7 (PMC6323827; doi:10.1186/s12864-018-5320-7)
Supplement: Supplementary file 9 — Figure S5. Changes in the reaction related to photosynthetic electron transport metabolism. (DOCX 206 kb) [file 12864_2018_5320_MOESM9_ESM.docx]

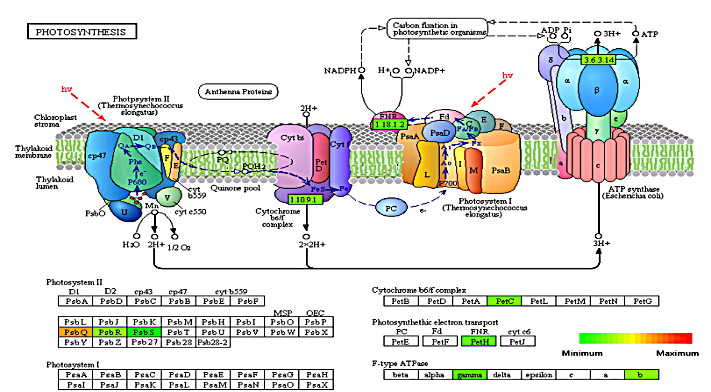


Figure S5. Changes in the reaction related to photosynthetic electron transport metabolism. The metabolism of each previously selected DEG was analyzed using the KEGG mapper.
