# Supplementary material for: Analysis of the distribution of assimilation products and the characteristics of transcriptomes in rice by submergence during the ripening stage
Source: BMC Genomics. 2019 Jan 8;20:18. doi: 10.1186/s12864-018-5320-7 (PMC6323827; doi:10.1186/s12864-018-5320-7)
Supplement: Supplementary file 13 — Figure S7. Changes in the reaction related to starch and sucrose metabolism in stems and leaves. (DOCX 277 kb) [file 12864_2018_5320_MOESM13_ESM.docx]

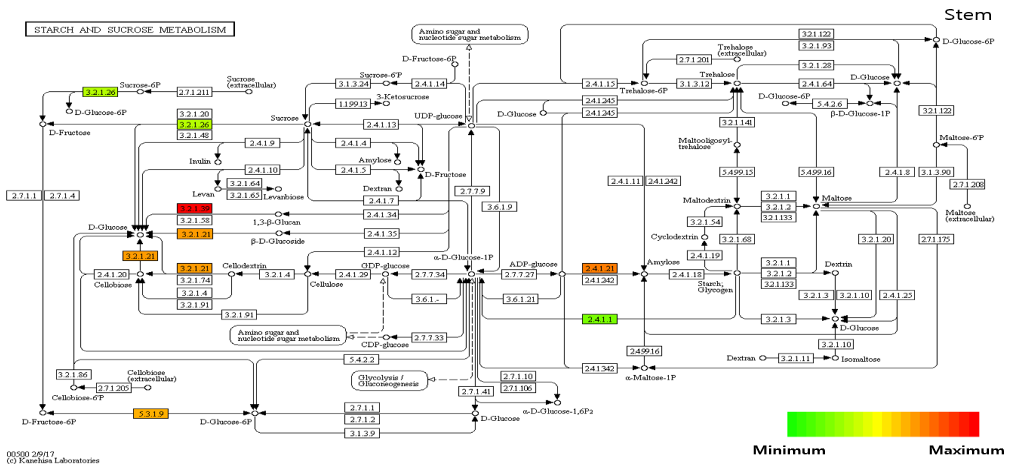


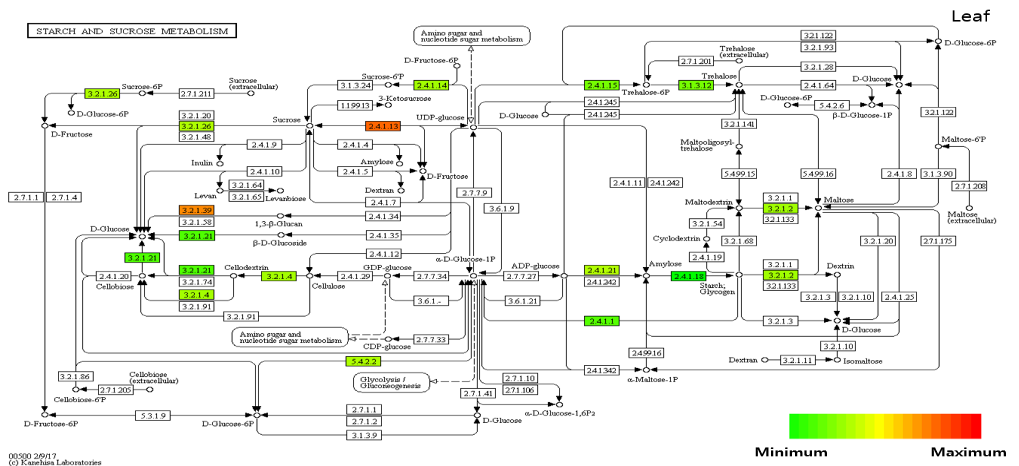


Figure S7. Changes in the reaction related to starch and sucrose metabolism in stems and leaves. The metabolism of each previously selected DEG was analyzed using the KEGG mapper.
