# Supplementary material for: Analysis of the distribution of assimilation products and the characteristics of transcriptomes in rice by submergence during the ripening stage
Source: BMC Genomics. 2019 Jan 8;20:18. doi: 10.1186/s12864-018-5320-7 (PMC6323827; doi:10.1186/s12864-018-5320-7)
Supplement: Supplementary file 15 — Table S1. Changes of water condition by submergence treatment during ripening period. (DOCX 16 kb) [file 12864_2018_5320_MOESM15_ESM.docx]

Table S1. Changes of water condition by submergence treatment during ripening period.

| Treatment | | DO  (mg/L) | BOD  (mg/L) | COD  (mg/L) | Suspended solids  (mg/L) | Turbidity  (NTU) |
| --- | --- | --- | --- | --- | --- | --- |
| Water quality | Hour |  |  |  |  |  |
| Clear  water | 0 | 8.0 | 2.9 | 12 | 654 | 245 |
|  | 6 | 7.8 | 2.8 | 9 | 667 | 264 |
|  | 12 | 7.7 | 2.9 | 17 | 879 | 221 |
|  | 24 | 7.4 | 3.3 | 24 | 1340 | 278 |
|  | 48 | 6.6 | 4.1 | 37 | 2793 | 301 |
|  | 72 | 5.1 | 4.2 | 36 | 2402 | 257 |
|  | 96 | 4.0 | 5.1 | 45 | 3450 | 298 |
| Muddy water | 0 | 7.2 | 3.3 | 20 | 921 | 1123 |
|  | 6 | 7.1 | 3.3 | 19 | 984 | 1262 |
|  | 12 | 6.9 | 3.5 | 42 | 1332 | 1103 |
|  | 24 | 6.1 | 4.5 | 58 | 2220 | 998 |
|  | 48 | 4.5 | 5.8 | 56 | 3440 | 1002 |
|  | 72 | 2.7 | 6.2 | 72 | 5420 | 887 |
|  | 96 | 2.1 | 7.9 | 88 | 4300 | 876 |

DO : Dissolved oxygen, BOD : Biochemical oxygen demand, COD : Chemical oxygen demand.
