# Supplementary material for: Analysis of the distribution of assimilation products and the characteristics of transcriptomes in rice by submergence during the ripening stage
Source: BMC Genomics. 2019 Jan 8;20:18. doi: 10.1186/s12864-018-5320-7 (PMC6323827; doi:10.1186/s12864-018-5320-7)
Supplement: Supplementary file 16 — Table S2. Gene specific PCR primer sets for quantitative RT-PCR amplification. (DOCX 17 kb) [file 12864_2018_5320_MOESM16_ESM.docx]

Table S2. Gene specific PCR primer sets for quantitative RT-PCR amplication.

| Enzyme | Gene  Name | Accession  No. | Sequence | Amplicon  Size |
| --- | --- | --- | --- | --- |
| Starch synthase  (soluble) | SS1 | AK109458 | F: GGGCCTTCATGGATCAACC  R: CCGCTTCAAGCATCCTCATC | 279 |
|  | SSIIa | AK101978 | F: GGCCAAGTACCAATGGTGAA  R: GCATGATGCATCTGAAACAAAGC | 272 |
| ADP-glucose pyrophosphorylase  (large subunit) | AGPL1 | AK100910 | F: ATGCAGTGCAGTGCGTCTTT  R: ACTTCACTCGGGGCAGCTTA | 183 |
| ADP-glucose pyrophosphorylase  (small subunit) | AGPS1 | AK073146 | F: AGAATGCTCGTATTGGAGAAAATG  R: GGCAGCATGGAATAAACCAC | 258 |
| Sucrose synthase | SuSy2 | AK072074 | F: TTCAGCAGGAGAAGCCGTCAGC  R: CCGGCGTTTATTTGAGGCAAGC | 150 |
| Granule bound Starch Synthase | GBSS1 | AK070431 | F: AACGTGGCTGCTCCTTGAA  R: TTGGCAATAAGCCACACACA | 218 |
| Starch Branching enzyme | SBE1 | AK065121 | F: TGGCCATGGAAGAGTTGGC  R: CAGAAGCAACTGCTCCACC | 191 |
| Sucrose transporter | SUT1 | AK100027 | F: AGTTCCGGTCGGTCAGCAT  R: ACCGAGGTGGCAACAAAG | 241 |
| Sucrose phosphate synthase | SPS1 | AK121341 | F: CTGGGATGGATTTCAGCAGT  R: GCTTTGACAAGGGTGGTGAT | 202 |
| Debranching enzyme | PUL | AK242137 | F:CTGTCCACACGAAGTGTCTA  R:TTGGACGCCTCGTATGCTGA | 120 |
